# Impact of Prior Infection on Severe Acute Respiratory Syndrome Coronavirus 2 Transmission in Syrian Hamsters

**DOI:** 10.3389/fmicb.2021.722178

**Published:** 2021-08-10

**Authors:** Cheng Zhang, Zhendong Guo, Nan Li, Huan Cui, Keyin Meng, Lina Liu, Li Zhao, Shanshan Zhang, Chengfeng Qin, Juxiang Liu, Yuwei Gao, Chunmao Zhang

**Affiliations:** ^1^Changchun Veterinary Research Institute, Chinese Academy of Agricultural Sciences, Changchun, China; ^2^College of Veterinary Medicine, Hebei Agricultural University, Baoding, China; ^3^College of Veterinary Medicine, Jilin University, Changchun, China; ^4^Beijing Institute of Microbiology and Epidemiology, Beijing, China

**Keywords:** severe acute respiratory syndrome coronavirus 2, prior infection, reinfection, contact transmission, airborne transmission

## Abstract

Prior infection with severe acute respiratory syndrome coronavirus 2 (SARS-CoV-2) provides protective immunity against reinfection. However, whether prior infection blocks SARS-CoV-2 transmission is not yet clear. Here, we evaluated the impact of prior infection on SARS-CoV-2 transmission in Syrian hamsters. Our results showed that prior infection significantly reduced SARS-CoV-2 replication in Syrian hamsters, but sterilizing immunity was not achieved. Prior infection blocked the airborne transmission of SARS-CoV-2 from previously infected Syrian hamsters to naïve Syrian hamsters and previously infected Syrian hamsters. Moreover, prior infection substantially reduced the efficiency of direct contact transmission between previously infected Syrian hamsters. However, prior infection had limited impact on SARS-CoV-2 transmission from previously infected Syrian hamsters to naïve Syrian hamsters *via* direct contact in the early course of infection. Human reinfection and SARS-CoV-2 transmission between a previously infected population and a healthy population would be likely, and a higher vaccination coverage rate was needed to reach herd immunity. Our work will aid the implementation of appropriate public health and social measures to control coronavirus infectious disease 2019 (COVID-19) pandemic.

## Introduction

Severe acute respiratory syndrome coronavirus 2 (SARS-CoV-2), the agent of coronavirus infectious disease 2019 (COVID-19), has severely disrupted healthcare system and economic activities in the world. At present, multiple vaccine candidates are in phase 3 clinical trials, and several of them have been approved for emergency use (Baden et al., 2020; Dai and Gao, 2020; Logunov et al., 2020; Polack et al., 2020; Zhang et al., 2020; Zhu et al., 2020; Ramasamy et al., 2021; Xia et al., 2021). Previous studies have shown that prior infection or vaccination provides protective immunity against SARS-CoV-2 in different animal models (Bosco-Lauth et al., 2020; Chandrashekar et al., 2020; Corbett et al., 2020; Deng et al., 2020; Gao et al., 2020; Mercado et al., 2020; van Doremalen et al., 2020; Wang et al., 2020a; Yu et al., 2020), but previously infected or vaccinated animals still shed large quantities of the virus in their upper respiratory tracts (Chandrashekar et al., 2020; Gao et al., 2020; van Doremalen et al., 2020; Yu et al., 2020).

Previously infected cats cannot be reinfected, while prior infected rhesus macaques cannot be reinfected either in the work of Deng et al. (2020) but can be reinfeted in Chandrashekar et al. (2020) (Bosco-Lauth et al., 2020). SARS-CoV-2 transmission study between prior infected rhesus macaques and the naïve animals was not found. Several human COVID-19 reinfection cases have been reported 1 year before (To et al., 2020; Van Elslande et al., 2020), even in the presence of neutralizing antibodies (Selhorst et al., 2020; Zhang et al., 2021). Hence, the recovered COVID-19 patients still need to follow public health measures and keep on personal protection. However, data about SARS-CoV-2 transmission from recovered COVID-19 patients to a healthy person was lacking. In principle, it may be possible. To find out the potential transmission from a reinfected person is very important for the government to make reasonable isolation and quarantine strategies, which would be good for saving resources and improving COVID-19 prevention and control. However, the impact of prior infection on SARS-CoV-2 transmission in humans is not clear.

Syrian hamsters have been used as a small animal model to study the pathogenesis (Chan et al., 2020; Imai et al., 2020) and transmission (Sia et al., 2020) of SARS-CoV-2 and evaluate vaccines (Sanchez-Felipe et al., 2020; Tostanoski et al., 2020) and antiviral drugs (Rosenke et al., 2020). Here, we evaluated the impact of prior infection on SARS-CoV-2 transmission in Syrian hamsters. Our results showed that prior infection substantially reduced SARS-CoV-2 transmission between previously infected Syrian hamsters, but direct contact transmission can still occur between previously infected hamsters and naïve Syrian hamsters. Our work will help governments and public health agencies make more reasonable public health decisions and aid the implementation of appropriate public health and social measures to control COVID-19.

## Materials and Methods

### Ethics and Biosecurity Statement

All animal experiments were approved by the Animal Care and Use Committee of Changchun Veterinary Research Institute. All experiments involving the infectious SARS-CoV-2 were performed in the Animal Biosafety Level 3 Laboratories of Changchun Veterinary Research Institute.

### Cells, Virus, and Infectious Viral Load Determination

Vero-E6 cells were grown in DMEM containing 10% fetus calf serum, 100 U/ml penicillin, and 100 μg/ml streptomycin. BetaCoV/Beijing/IME-BJ05-2020 was isolated and propagated in Vero-E6 cells, and the virus stock was titrated into TCID_50_/ml. Serial 10-fold dilutions of the samples were added to Vero-E6 cells with 80% confluence in 96-well plates, and incubated for 4 days at 37°C with 5% CO_2_. The cytopathic effect was observed under a microscopy. The viral titers were determined by the Reed-Muench method. The viral titer of the stock is 10^7.0^ TCID_50_/ml.

### SARS-CoV-2 Challenge Studies

As shown in Supplementary Figure S1, 12 male Syrian hamsters, 4–5 weeks old, were anesthetized with isoflurane and intranasally inoculated with 10^5^ TCID_50_ of SARS-CoV-2. Four Syrian hamsters were sacrificed for serum samples at 21 days post infection (dpi), and other eight animals were rechallenged with 10^6^ TCID_50_ of the virus. As the infected control (IC), another six naïve Syrian hamsters were inoculated with 10^6^ TCID_50_ of the virus. At 2 and 4 dpi, half animals in each group were sacrificed for nasal turbinates and lungs. The supernatants of the homogenized nasal turbinates and lungs were used for virus titration in Vero-E6 cells and for viral RNA quantification using real-time RT-qPCR.

### SARS-CoV-2 Transmission Studies

At 21 days after intranasal inoculation with 10^5^ TCID_50_ of SARS-CoV-2, these male Syrian hamsters were used for SARS-CoV-2 transmission studies. The direct contact transmission and airborne transmission experiments were usually performed at 24 h after inoculation. Considering the rapid clearance of virus in previously infected animals, we also performed the transmission experiments at 2 h after inoculation to find out potential transmission in the early infection stage. Three animals in each group were initially used in the transmission experiments, and later more animals were allowed, and four Syrian hamsters in each group were used in direct contact transmission. To test direct contact transmission, three or four donor Syrian hamsters were intranasally inoculated with 10^6^ TCID_50_ of SARS-CoV-2. Around 2 or 24 h after inoculation, another three or four recipient animals were transferred to a new cage and cohoused together with the donor animals. At 1, 3, 5, and 7 days post exposure (dpe), nasal washes were collected from all animals by intranasal infusion with 1 ml PBS. To test the airborne transmission, three donor Syrian hamsters were inoculated with 10^6^ TCID_50_ of SARS-CoV-2. About 2 or 24 h after inoculation, the three donors and another three recipient animals were transferred to an airborne transmission cage, which was equipped with two-wire-mesh partitions that prevent direct contact between animals and allow the spread of SARS-CoV-2 *via* the flow air. The distance between the two-wire-mesh partitions is 2 cm. Air flows from the donor cage to the cage housing the recipients. Nasal washes were collected from all animals by intranasal infusion with 1 ml PBS at 1, 3, 5, and 7 dpe. For transmission of the virus from initially infected naïve Syrian hamsters to previously infected Syrian hamsters, the naïve Syrian hamsters were used as the donors and previously infected animals were used as the recipients (Supplementary Figure S2). Inversely, for transmission of the virus from previously infected Syrian hamsters to the naïve Syrian hamsters, previously infected animals were used as the donors and the naïve animals were used as the recipients (Supplementary Figure S3). For transmission of the virus between previously infected hamsters, randomly selected previously infected animals were used as the donors and the recipients (Supplementary Figure S4). For the lower dose inoculation on SARS-CoV-2 transmission, the donor animals were inoculated with 10^4^ TCID_50_ of SARS-CoV-2, and then treated similarly as animals in other transmission experiments (Supplementary Figure S5).

### Viral RNA Quantification

RNA was extracted from 200 μl samples using the viral RNA minikits (QIAGEN, Hilden, Germany) and eluted with 90 μl water, and 15 μl RNA was used for the real-time qPCR to detect the N gene of SARS-CoV-2 using the Detection Kits for 2019-Novel Coronavirus RNA (Shenzhen Puruikang Biotech, China). The experiments were performed with an ABI7500 system (Roche, Switzerland). The amplification reaction conditions were 50°C for 20 min for reverse transcriptase, followed by 95°C for 3 min, and then 45 cycles of 95°C for 5 s, 57°C for 45 s, and finally 25°C for 10 min. The viral RNA copies were estimated from the measured cycle threshold (Ct) values.

### Viral Subgenomic RNA Quantification

The nasal turbinates and lungs were homogenized and lysed in 1 ml of the buffer RLCK (QIAGEN, Hilden, Germany) for 10 min. RNA was extracted from 500 μl of the centrifuged supernatants and eluted with 90 μl water. A pair of primers and a Taqman probe was designed targeting the E gene sgmRNA of SARS-CoV-2 (Wolfel et al., 2020). About 9 μl RNA was used for real-time qPCR to detect the E gene sgmRNA using the One Step PrimeScriptTM III RT-qPCR Mix (Code No: RR600A, Takara). The reaction conditions were 52°C for 5 min for reverse transcriptase, followed by 95°C for 10 s, and then 45 cycles of 95°C for 5 s, 60°C for 30 s. The sgmRNA copies were estimated from the measured cycle threshold (Ct) values.

### Virus Neutralization Assay

Around 100 μl of virus dilutions, containing 100 TCID_50_ of SARS-CoV-2, was incubated with 100 μl 2-fold serial dilutions of the infected Syrian hamster serum or mock serum for 1 h, and 200 μl of the mixtures were added to Vero-E6 cells with 80% confluence in 96-well plates, and incubated for 4 days at 37°C with 5% CO_2_. The cytopathic effects were observed under a microscopy. Each sample has three replicates. The neutralizing antibody titers (NT_100_) were determined as the reciprocal number of the highest serum dilution that completely prevented the cytopathic effect. A serum sample with neutralizing antibody titer <1:10 was considered to be negative.

### Statistics Analysis

The unpaired *t*-test was used to analyze the significant differences of viral titers, RNA copies, and sgmRNA copies in nasal turbinates and lungs between different groups [*p* > 0.05, not significant (ns); ^*^p < 0.05; ^**^p < 0.01; and ^***^p < 0.001]. All data was analyzed with the software GraphPad Prism 6.02.

## Results

### Prior Infection Reduced SARS-CoV-2 Replication in Syrian Hamsters

**Figure 1:**
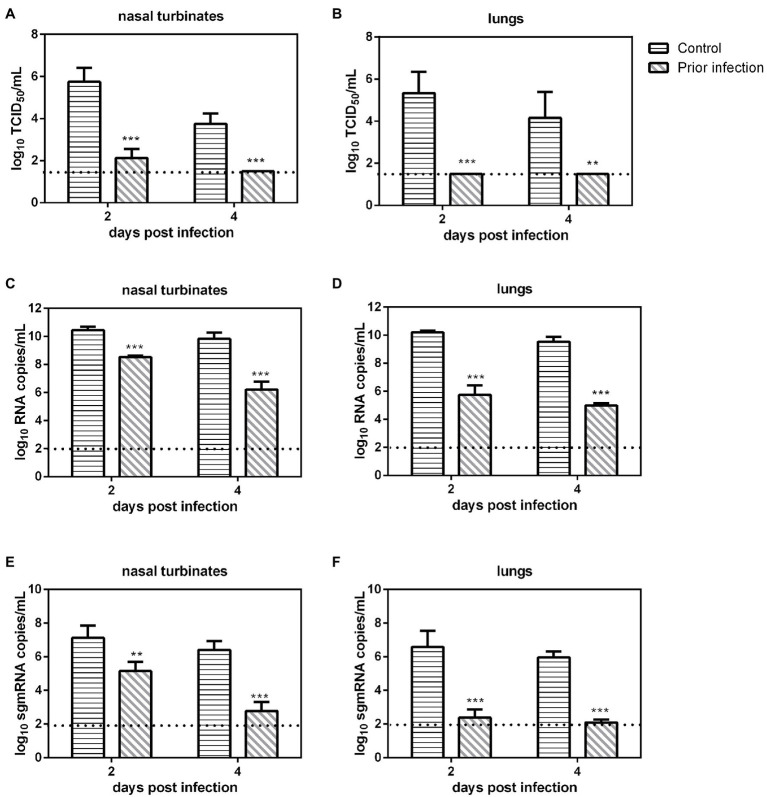
Viral load in previously infected Syrian hamsters intranasally inoculated with severe acute respiratory syndrome coronavirus 2 (SARS-CoV-2). The dotted line represents the detection limit of viral titers and RNA copies. **(A,B)** Viral titers (log_10_TCID_50_/ml) detected in the nasal turbinates **(A)** and lungs **(B)** of previously infected Syrian hamsters challenged with SARS-CoV-2. **(C,D)** Viral RNA copies (log_10_RNA copies/ml) detected in the nasal turbinates **(C)** and lungs **(D)** of previously infected Syrian hamsters challenged with SARS-CoV-2. **(E,F)** Viral sgmRNA copies (log_10_sgmRNA copies/ml) detected in the nasal turbinates **(E)** and lungs **(F)** of previously infected Syrian hamsters challenged with SARS-CoV-2. ^**^p < 0.01; ^***^p < 0.001.

We evaluated SARS-CoV-2 replication in previously infected Syrian hamsters. Serum samples were collected at 21 dpi from inoculated Syrian hamsters, and then these animals were challenged with 10^6^ TCID_50_ of SARS-CoV-2, and nasal turbinates and lungs were collected at 2 and 4 dpi for viral load determination. The virus neutralization assay revealed that all serum samples of previously infected Syrian hamsters had very high neutralizing antibody titers (1:2560–1:5120). In the nasal turbinates, the viral loads, the number of RNA copies and sgmRNA copies in the IC group at 2 and 4 dpi were significantly higher than those in prior infection group (Figures 1A,C,E). In the lungs, the viral load, the numbers of RNA copies and sgmRNA copies in the IC group at 2 and 4 dpi were also significantly higher than those in prior infection group (Figures 1B,D,F). In summary, prior infection substantially reduced SARS-CoV-2 replication in previously infected Syrian hamsters.

### SARS-CoV-2 Transmission From Initially Infected Syrian Hamsters to Previously Infected Syrian Hamsters

**Figure 2:**
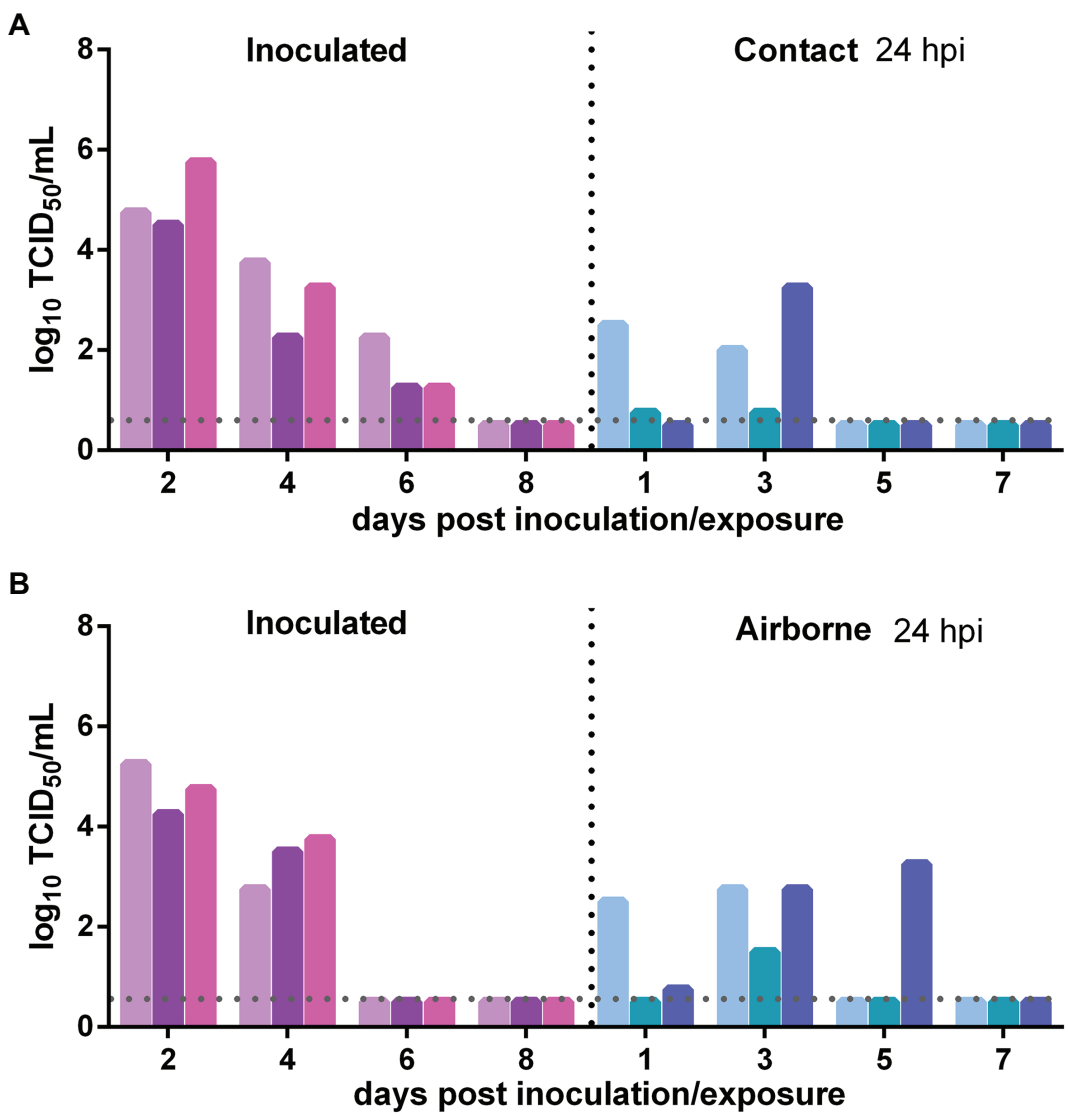
Transmission of SARS-CoV-2 from initially infected Syrian hamsters to previously infected Syrian hamsters 24 h post inoculation (hpi). The dotted line represents the detection limit of viral titers and colored bars are indicating individual animals. The time between infection and start of the transmission experiment was showed on the upper right side. **(A)** Viral titers detected in the nasal washes of the donors inoculated with 10^6^ TCID_50_ of SARS-CoV-2 and previously infected contacts. **(B)** Viral titers detected in the nasal washes of donors inoculated with SARS-CoV-2 and previously infected recipients in the airborne transmission group.

The direct contact transmission and airborne transmission experiments were performed at 24 h after inoculation with 10^6^ TCID_50_ of the virus. As to direct contact transmission, viral titers in the nasal washes of the donor Syrian hamsters were maintained at a high level at 2 dpi and then declined rapidly (Figure 2A). In the contacts, at 1 day post exposure (dpe), live SARS-CoV-2 was detected in the nasal washes of two previously infected contacts. At 3 dpe, live virus was detected in all three previously infected contacts (Figure 2A). Therefore, the virus was efficiently transmitted from the inoculated donors to the previously infected contacts. As to airborne transmission, live SARS-CoV-2 was detected in the nasal washes of two previously infected recipient Syrian hamsters at 1 dpe, and at 3 dpe, all three previously infected recipients had been infected by SARS-CoV-2, and the viral titers in the nasal washes were significantly increased (Figure 2B). At 5 dpe, the viral titer in one previously infected recipient was still high (10^3.25^ TCID_50_/ml; Figure 2B). Therefore, the virus was efficiently transmitted from the inoculated donors to previously infected recipients by the airborne route. In summary, SARS-CoV-2 was efficiently transmitted from the inoculated donors to previously infected Syrian hamsters by direct contact and airborne transmission.

### SARS-CoV-2 Transmission From Previously Infected Syrian Hamsters to Naïve Syrian Hamsters

**Figure 3:**
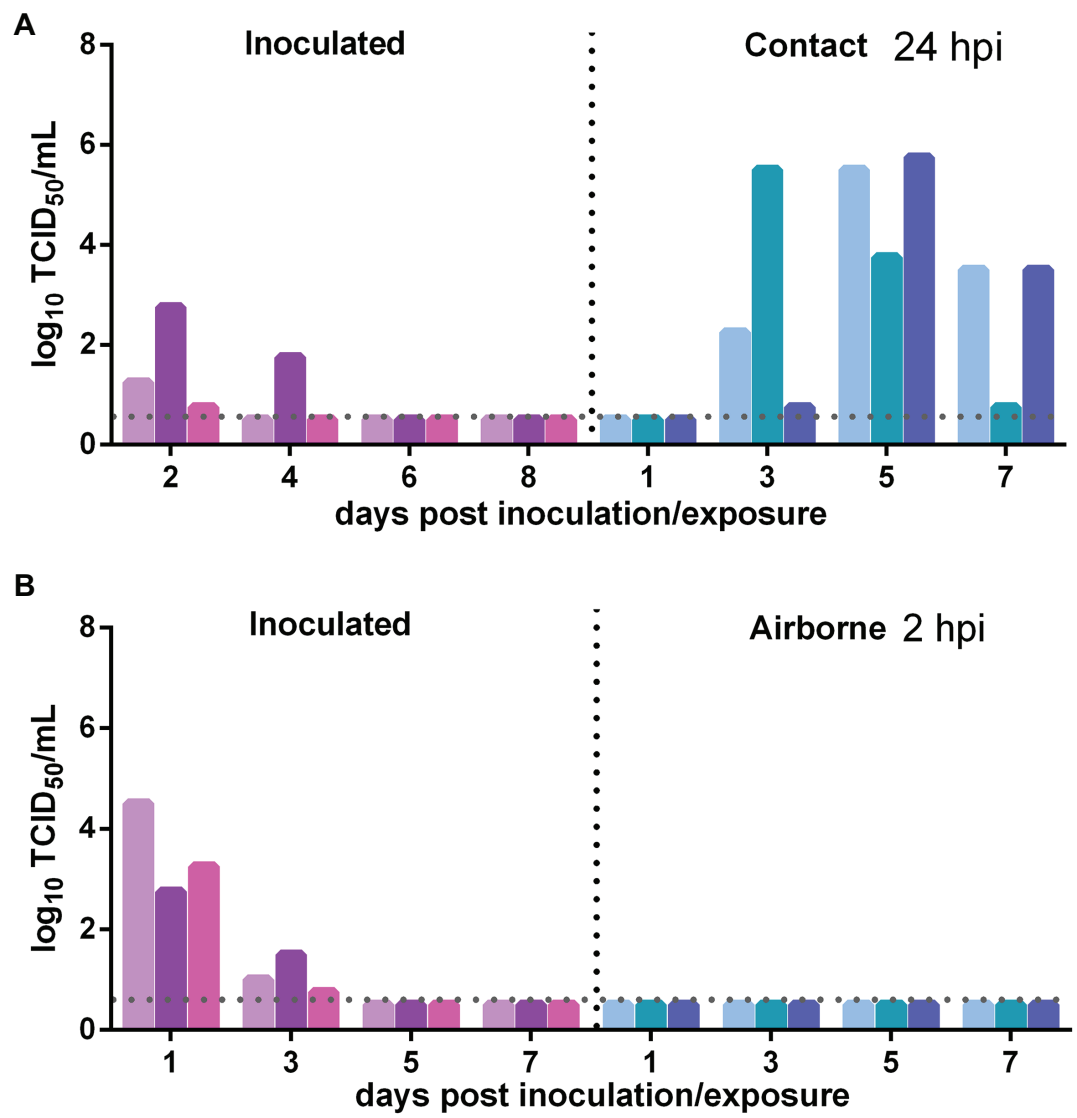
Transmission of SARS-CoV-2 from previously infected Syrian hamsters to uninfected Syrian hamsters 24 hpi. The dotted line represents the detection limit of viral titers and colored bars are indicating individual animals. The time between infection and start of the transmission experiment was showed on the upper right side. **(A)** Viral titers detected in the nasal washes of previously infected donors inoculated with SARS-CoV-2 and the naïve contacts in the contact transmission experiment performed at 24 h after inoculation. **(B)** Viral titers detected in the nasal washes of previously infected donors inoculated with SARS-CoV-2 and the naïve recipients in the airborne transmission experiment performed at 2 h after inoculation.

The direct contact and airborne transmission experiments were first performed at 24 h after inoculation. As to direct contact transmission, at 2 dpi, the viral titers in the nasal washes of two donors were very low and that in the third donor animal was moderate (10^2.75^ TCID_50_/ml), and at 4 dpi, live virus was not detected in two donors, and the third donor had a very low viral titer (Figure 3A). At 3 dpe, live virus was detected in the nasal washes of all three contacts, and at 5 dpe, the viral titers in all contacts were very high (Figure 3A). Viral neutralizing antibodies were detected in the serum of all contacts at 14 dpe, with titers from 1:640 to 1:1,280. Therefore, the virus was efficiently transmitted from previously infected donors to naïve contacts by direct contact. The airborne transmission experiment was performed 2 h after inoculation. A moderate virus titer was detected at 1 and 3 dpi in the previously infected donors, and no live virus was still detected in any naïve recipients in the airborne transmission group (Figure 3B). No neutralizing antibodies (<1:10) were detected in the serum of the recipients in these two airborne transmission groups at 14 dpe. Therefore, the virus was not transmitted from previously infected donors to naïve recipients by the airborne route. In summary, SARS-CoV-2 was effectively transmitted from previously infected donors to naïve Syrian hamsters by direct contact, but not by airborne transmission.

### SARS-CoV-2 Transmission Between Previously Infected Syrian Hamsters

**Figure 4:**
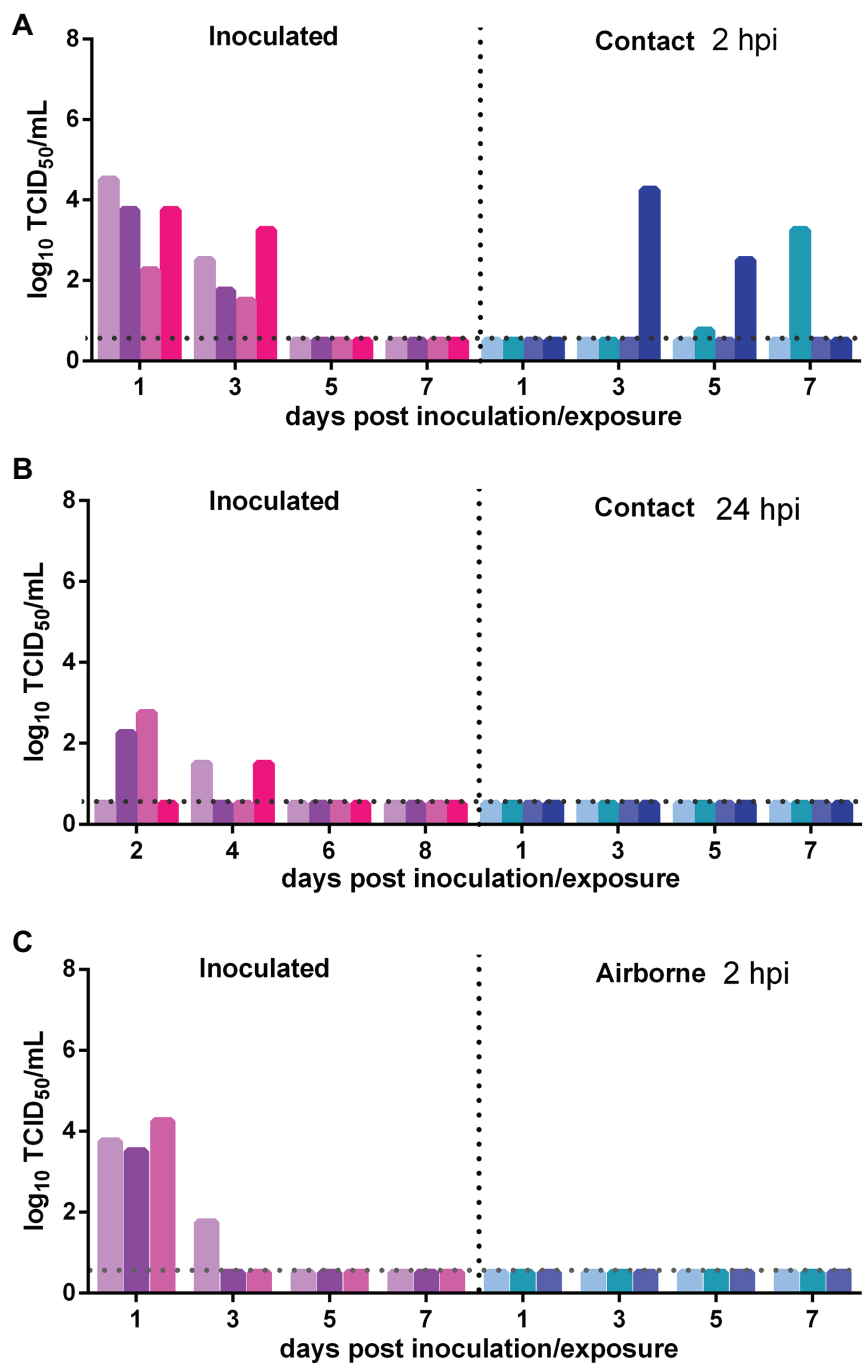
Transmission of SARS-CoV-2 between previously infected Syrian hamsters. The dotted line represents the detection limit of the viral titer and colored bars are indicating individual animals. The time between infection and start of the transmission experiment was showed on the upper right side. **(A)** Viral titers detected in the nasal washes of previously infected donors inoculated with SARS-CoV-2 and previously infected contacts in the contact transmission experiment performed at 2 hpi. **(B)** Viral titers detected in the nasal washes of previously infected donors inoculated with SARS-CoV-2 and previously infected contacts in the contact transmission experiment performed at 24 hpi. **(C)** Viral titers detected in the nasal washes of previously infected donors inoculated with SARS-CoV-2 and previously infected recipients in the airborne transmission experiment performed at 2 hpi.

The direct contact and airborne transmission experiments were first performed at 2 h after inoculation. As to direct contact transmission, high viral titers were detected in the nasal washes of the donor Syrian hamsters at 1 and 3 dpi (10^3.56^ TCID_50_/ml and 10^2.25^ TCID_50_/ml; Figure 4A). At 3 dpe, live virus was detected in the nasal washes of one contact Syrian hamster (10^4.25^ TCID_50_/ml), and at 5 dpe, live virus was detected in a second contact (10^0.75^ TCID_50_/ml), and at 7 dpe, the virus titer in this contact animal had substantially increased to 10^3.25^ TCID_50_/ml (Figure 4A). The direct contact transmission experiment was similarly performed 24 h after inoculation. A moderate virus titer was detected in two donors, and no live virus was detected in the nasal washes of any contact animal (Figure 4B). Therefore, the virus was transmitted between previously infected Syrian hamsters by direct contact during a very short period early in the course of the infection. As to airborne transmission, high viral titers were detected in the donors at 1 dpi, and no live virus was detected in the nasal washes of any recipients in the airborne transmission group (Figure 4C). Therefore, the virus was not transmitted between previously infected Syrian hamsters by the airborne route. In summary, SARS-CoV-2 has limited transmission ability between previously infected Syrian hamsters by direct contact during a short period early in the course of infection but not by the airborne route.

### Impact of a Lower Inoculation Dose on SARS-CoV-2 Transmission

**Figure 5:**
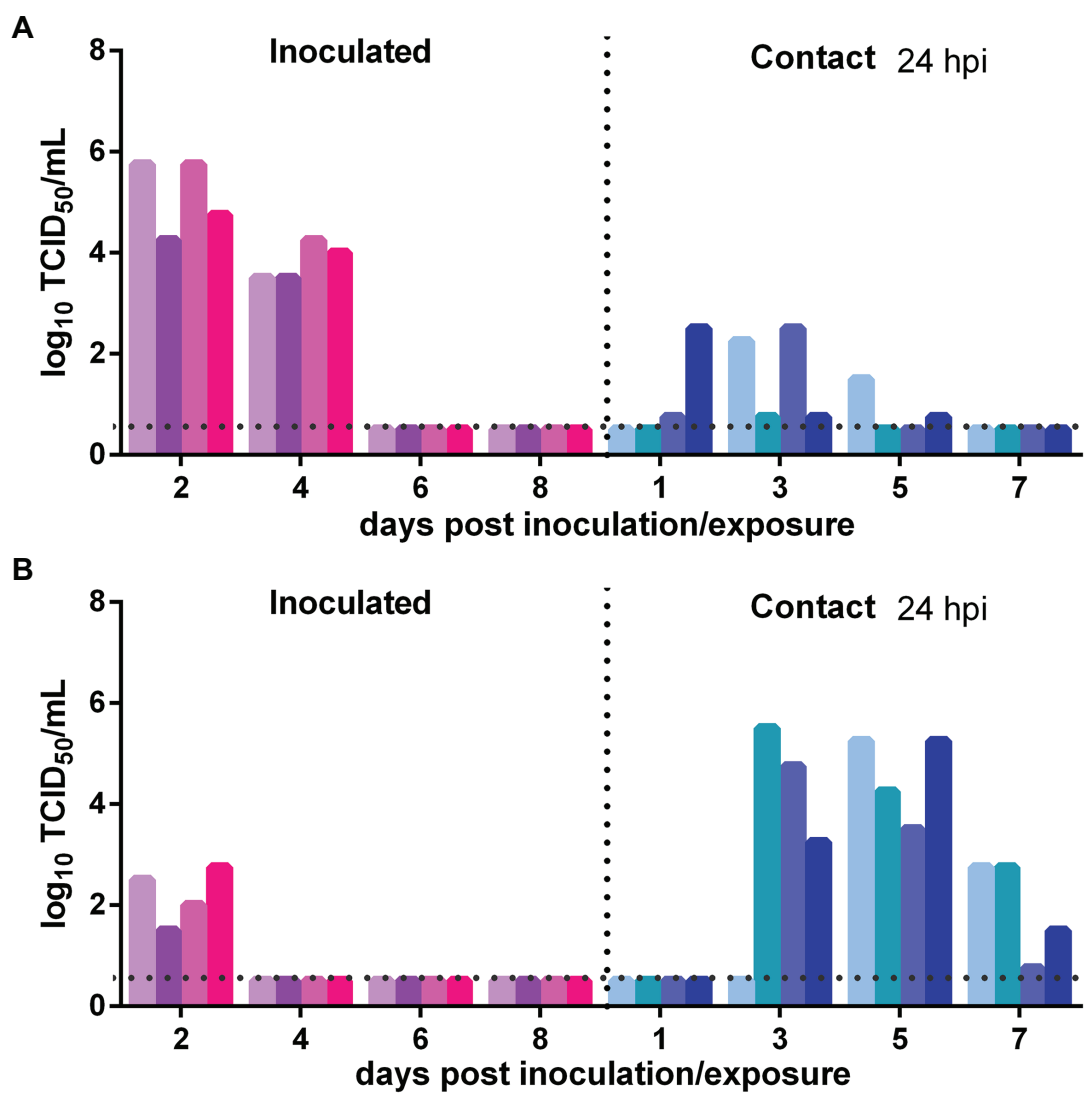
Impact of a lower inoculation dose on SARS-CoV-2 transmission between naïve Syrian hamsters and previously infected Syrian hamsters at 24 hpi with 10^4^ TCID_50_ of SARS-CoV-2. The dotted line represents the detection limit of the viral titer and colored bars are indicating individual animals. The time between infection and start of the transmission experiment was showed on the upper right side. **(A)** Viral titers detected in the nasal washes of the initially infected donors inoculated with SARS-CoV-2 and the previously infected contacts. **(B)** Viral titers detected in the nasal washes of previously infected donors inoculated with SARS-CoV-2 and the naïve contacts.

The direct contact transmission experiments were performed at 24 h after inoculation with 10^4^ TCID_50_ of the virus. As to the transmission of the virus from the initially inoculated naïve donor animals to previously infected contacts, the viral titers in the nasal washes of the donors were maintained at a high level at 2 and 4 dpi and then rapidly declined (Figure 5A). At 1 dpe, live virus was detected in two previously infected contacts, and at 3 dpe, live virus was found in the nasal washes of all four previously infected contacts (Figure 5A). Therefore, the virus was efficiently transmitted from the inoculated donors to previously infected contacts by direct contact. As to the transmission of the virus from previously infected animals to naïve Syrian hamsters, at 2 dpi, the viral titer in the nasal washes of the donors was at a moderate level (10^2.25^ TCID_50_/ml; Figure 5B). At 3 dpe, live virus was detected in the nasal washes of three of the four contacts, and by 5 dpe, the fourth contact had also been infected by SARS-CoV-2 (Figure 5B). Therefore, the virus was efficiently transmitted from previously infected donors to naïve contacts. In summary, with a lower inoculation dose of 10^4^ TCID_50_ of the virus, SARS-CoV-2 was efficiently transmitted between previously infected Syrian hamsters and naïve Syrian hamsters by direct contact.

## Discussion

Our study showed that prior infection with SARS-CoV-2 substantially reduced viral replication in Syrian hamsters. However, sterilizing immunity was not achieved, and the virus still replicated at a moderate level in the nasal turbinates of previously infected animals. Moreover, we first showed that previously infected Syrian hamsters can be naturally reinfected by direct contact or the airborne route in an intense reexposure setting, but with lower viral titers in upper respiratory tract. This conclusion is consistent with recent reports showing that recovered COVID-19 patients were reinfected in the presence of neutralizing antibodies (Selhorst et al., 2020; Zhang et al., 2021), and low levels of subgenomic RNA was detected in the nasal swabs of previously infected rhesus macaques when rechallenge (Chandrashekar et al., 2020). Different from our results, several recent studies reported that prior infection in COVID-19 patients was highly protective against SARS-CoV-2 reinfection, with protective efficiency from 81.4 to 95.2% (Abu-Raddad et al., 2020, 2021b; Rennert and McMahan, 2021; Sheehan et al., 2021). This may be attributed to the differences in exposure time and doses. In our study, the exposure time was much longer than that of recovered COVID-19 patients in everyday life, and the exposure dose was also much higher than that of those patients, which might lead to a relatively high reinfection rate in previously infected Syrian hamsters. A cohort study of 175 recovered COVID-19 patients revealed that 6% of COVID-19 patients did not show any antibody response at all, and 30% of COVID-19 patients had very low levels of neutralizing antibodies (Wu et al., 2020). Considering the gradual decay of neutralizing antibodies (Ibarrondo et al., 2020; Muecksch et al., 2020; Wang et al., 2020b) and a considerable recovered COVID-19 patients with very low levels of neutralizing antibodies and the occurring variants, some human reinfections would be unavoidable. Indeed, a recent report showed 2.2% of previously infected students were reinfected during the Spring 2021 semester (Rennert and McMahan, 2021).

We also showed that prior infection blocked the airborne transmission of SARS-CoV-2 from previously infected Syrian hamsters to naïve Syrian hamsters and previously infected Syrian hamsters and substantially reduced the efficiency of direct contact transmission between previously infected Syrian hamsters. We believed that prior infection with SARS-CoV-2 would provide direct protection for those COVID-19 patients themselves and substantial indirect protection for those healthy people. However, prior infection had limited impact on SARS-CoV-2 transmission from previously infected Syrian hamsters to naïve Syrian hamsters *via* direct contact in the early course of infection. It was also true even at a lower inoculation dose of 10^4^ TCID_50_ of the virus (Figure 5). The potential transmission from the prior infected to a healthy population should not be neglected.

Given the potential reinfection and transmission between the previously infected and uninfected, and waning immunity in recovered COVID-19 patients (Ibarrondo et al., 2020; Muecksch et al., 2020), and immune evasion of the occurring variants, such as Alpha (B.1.1.7), Beta (B.1.351), Gamma (P.1), and Delta (B.1.617.2) of SARS-CoV-2, it would be much more difficult than initially believed to achieve herd immunity by natural infection or vaccination. A correspondence from Qatar showed that the protective efficiency of the mRNA vaccine BNT162b2 was dropped from 95% against COVID-19 to 72% against Beta (B1.351) variants (Abu-Raddad et al., 2021a). We think that a higher vaccination coverage rate is needed to reach herd immunity. However, it is worth emphasizing that there are important differences between SARS-CoV-2 infection and transmission in humans and Syrian hamsters, and our work should be interpreted with caution. At present, many governments are considering introducing immunity passports to help with the recovery of social and economic activities (Phelan, 2020), but the evidence supporting this proposal is insufficient. How does vaccination with COVID-19 vaccines impact the transmission of SARS-CoV-2 and its variants in humans? Data is still lacking and there is no clear answer. Further studies evaluating the efficiency of different vaccines with regard to blocking SARS-CoV-2 transmission *via* different routes in humans and animal models are urgently needed.

## Data Availability Statement

The original contributions presented in the study are included in the article/Supplementary Material, further inquiries can be directed to the corresponding authors.

## Ethics Statement

The animal study was reviewed and approved by the Animal Care and Use Committee of Changchun Veterinary Research Institute.

## Author Contributions

CuZ and YG designed the project. CuZ, CeZ, ZG, NL, HC, LL, LZ, KM, and SZ performed the experiments. CuZ, CeZ, ZG, and NL analyzed the data. CuZ drafted the manuscript. YG, CQ, JL, and ZG critically revised the manuscript. All authors contributed to the article and approved the submitted version.

## Conflict of Interest

The authors declare that the research was conducted in the absence of any commercial or financial relationships that could be construed as a potential conflict of interest.

## Publisher’s Note

All claims expressed in this article are solely those of the authors and do not necessarily represent those of their affiliated organizations, or those of the publisher, the editors and the reviewers. Any product that may be evaluated in this article, or claim that may be made by its manufacturer, is not guaranteed or endorsed by the publisher.
