# Supplementary material for: Impact of Prior Infection on Severe Acute Respiratory Syndrome Coronavirus 2 Transmission in Syrian Hamsters
Source: Front Microbiol. 2021 Aug 10;12:722178. doi: 10.3389/fmicb.2021.722178 (PMC8383181; doi:10.3389/fmicb.2021.722178)
Supplement: Supplementary file 1 [file Data_Sheet_1.docx]

**Supplementary Information for**

Impact of Prior Infection on SARS-CoV-2 Transmission in Syrian Hamsters

Cheng Zhang^1, 2&^, Zhendong Guo^1&^, Nan Li^1&^, Huan Cui^1, 3^, Keyin Meng^1^, Lina Liu^1^, Li Zhao^1^, Shanshan Zhang^1^, Juxiang Liu^2^, Chengfeng Qin^4^, Yuwei Gao^1#^, Chunmao Zhang^1#^

^1^Changchun Veterinary Research Institute, Chinese Academy of Agricultural Sciences, Changchun, China

^2^ College of Veterinary Medicine, Hebei Agricultural University, Baoding, China

^3^ College of Veterinary Medicine, Jilin University, Changchun, China

^4^ Beijing Institute of Microbiology and Epidemiology, Beijing, China

^&^these authors contributed equally.

^#^ Correspondence author:

Chunmao Zhang: [jk704715@sina.com](mailto:jk704715@sina.com)

Yuwei Gao: [gaoyuwei@gmail.com](mailto:gaoyuwei@gmail.com)

Figures


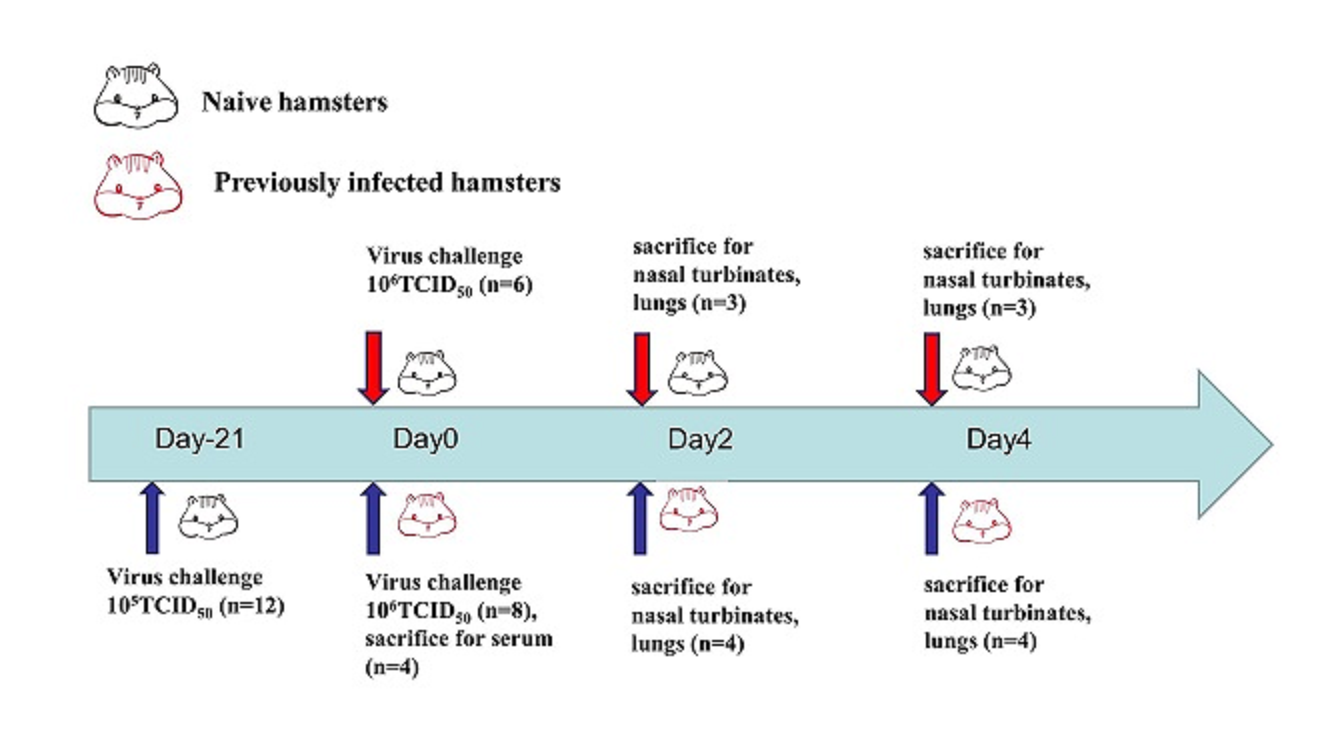


**Figure S1** The flowchart of the SARS-CoV-2 challenge study. Twelve male Syrian hamsters were intranasally inoculated with 10^5^ TCID_50_ of SARS-CoV-2 at 21 days ago. At day0, four Syrian hamsters were sacrificed for serum samples, and other eight previously infected hamsters were challenged with 10^6^ TCID_50_ of the virus. As the infected control (IC), six naïve Syrian hamsters were inoculated with 10^6^ TCID_50_ of the virus at day 0. Then at 2 and 4 days post infection, half animals in each group were sacrificed for nasal turbinates and lungs.


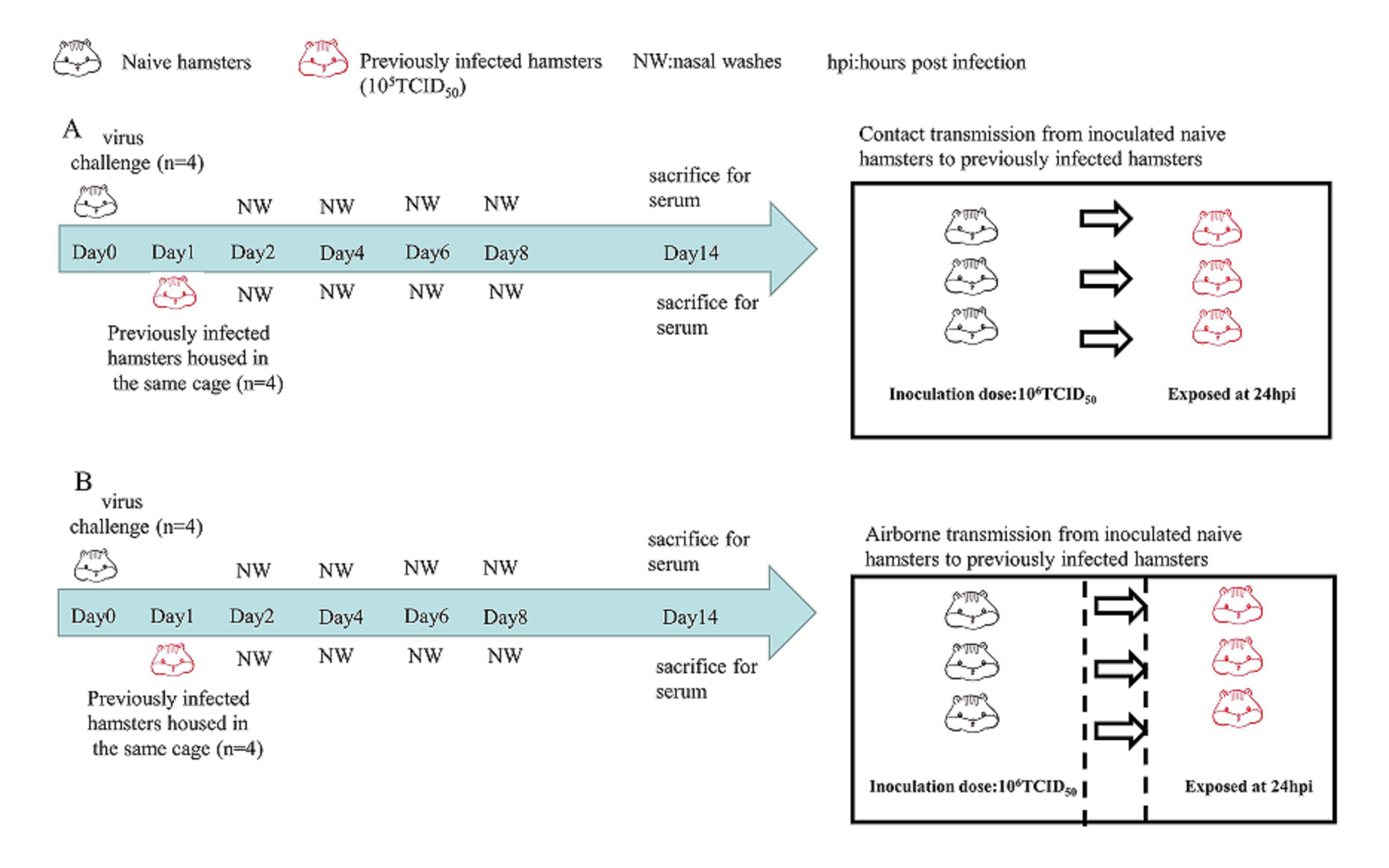


**Figure S2** The flowchart of SARS-CoV-2 transmission from initially infected Syrian hamsters to previously infected Syrian hamsters at 24 hours post infection. (A) SARS-CoV-2 contact transmission from initially infected Syrian hamsters (n=3) to previously infected Syrian hamsters (n=3). (B) SARS-CoV-2 airborne transmission from initially infected Syrian hamsters (n=3) to previously infected Syrian hamster (n=3).


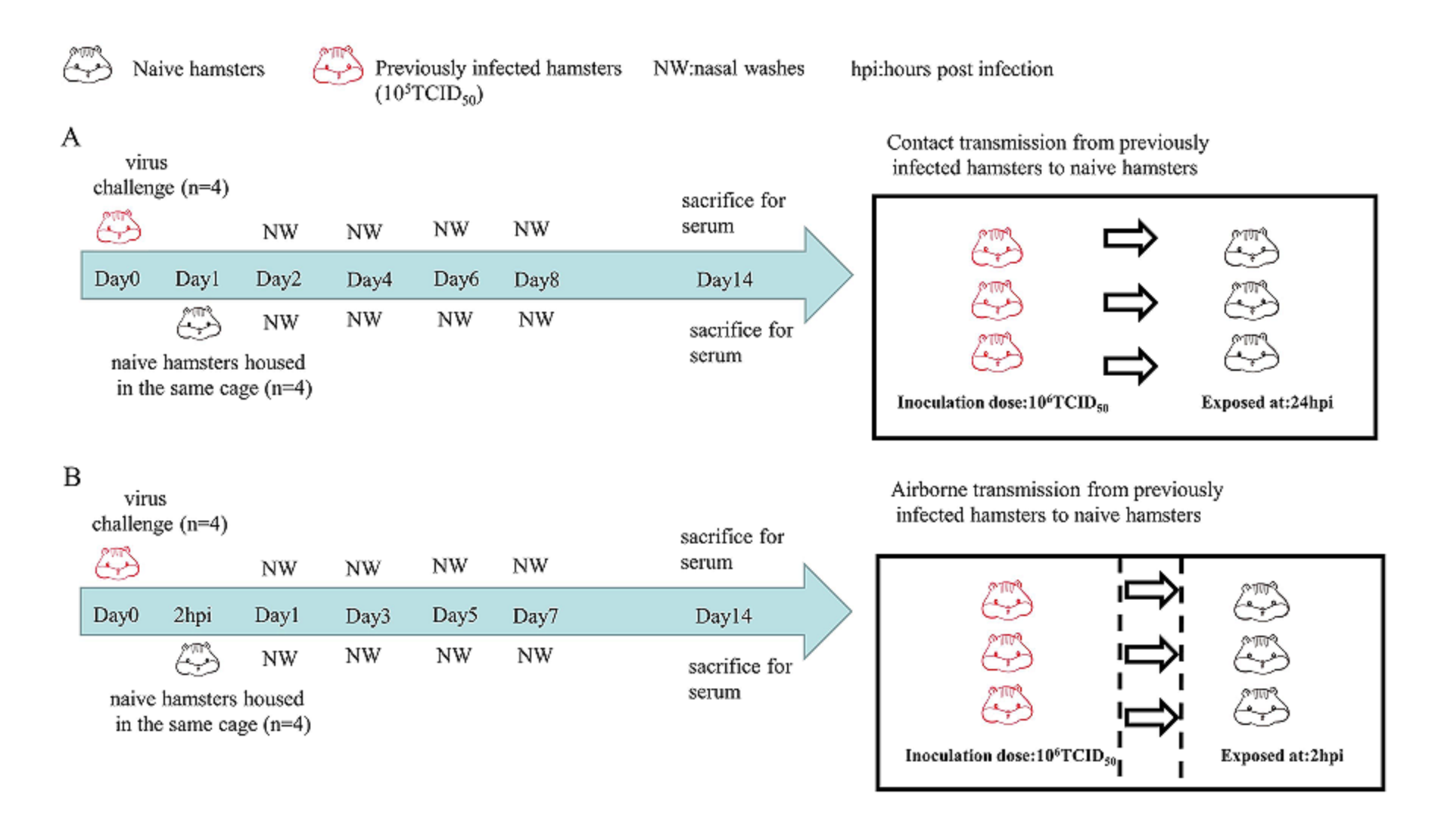


**Figure S3** The flowchart of SARS-CoV-2 transmission from previously infected hamsters to naïve

Syrian hamsters. (A) SARS-CoV-2 contact transmission from previously infected Syrian hamsters (n=3) to naïve Syrian hamsters (n=3) 24 hours after inocualtion. (B) SARS-CoV-2 airborne transmission from previously infected Syrian hamsters (n=3) to naïve Syrian hamsters (n=3) two hours after inoculation.


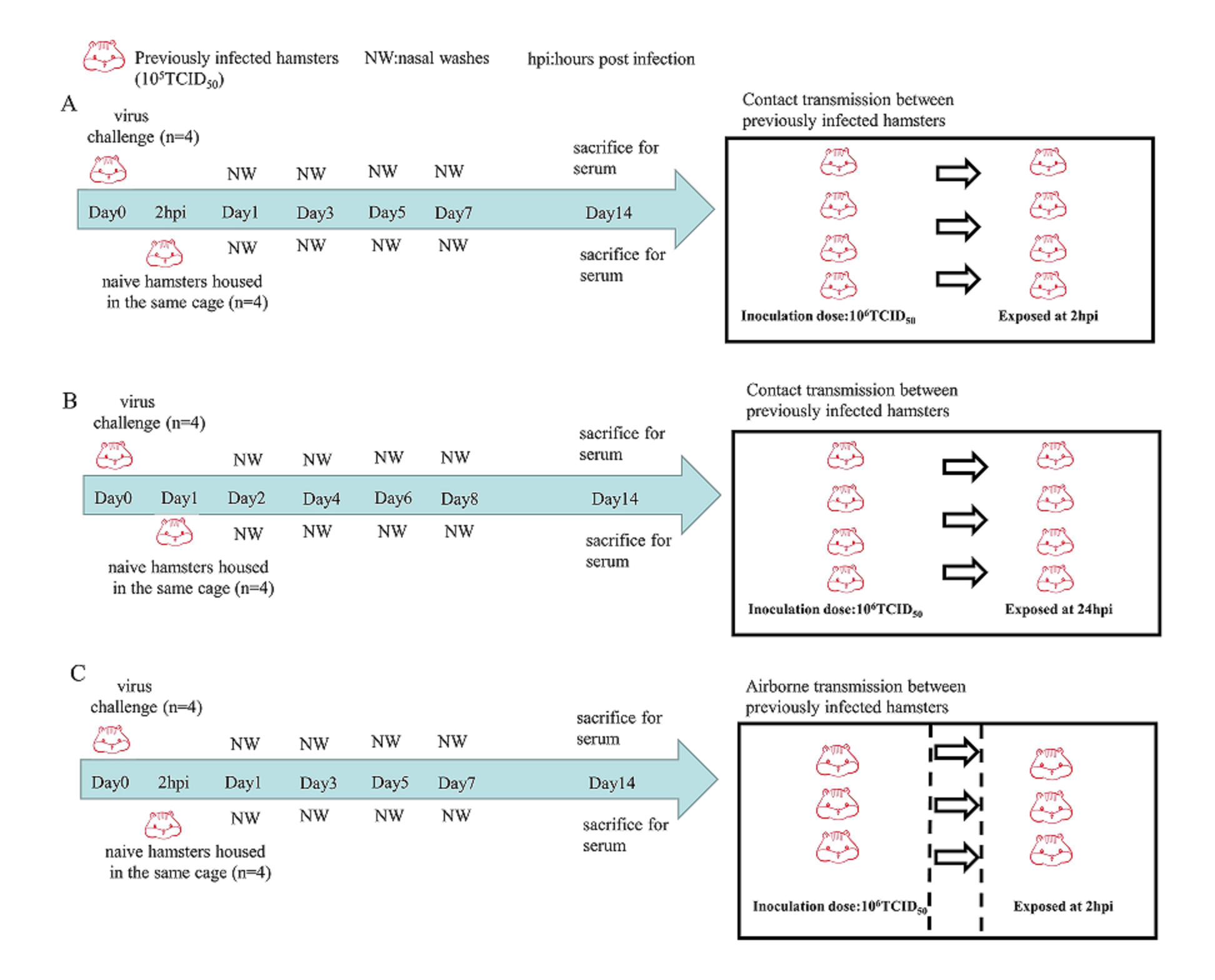


**Figure S4** The flowchart of SARS-CoV-2 transmission between previously infected Syrian hamsters. (A) SARS-CoV-2 contact transmission between previously infected Syrian hamsters (n=4) two hours after inoculation. (B) SARS-CoV-2 contact transmission between previously infected Syrian hamsters (n=4) 24 hours after inoculation. (C) SARS-CoV-2 airborne transmission between previously infected Syrian hamsters (n=4) two hours after inoculation.


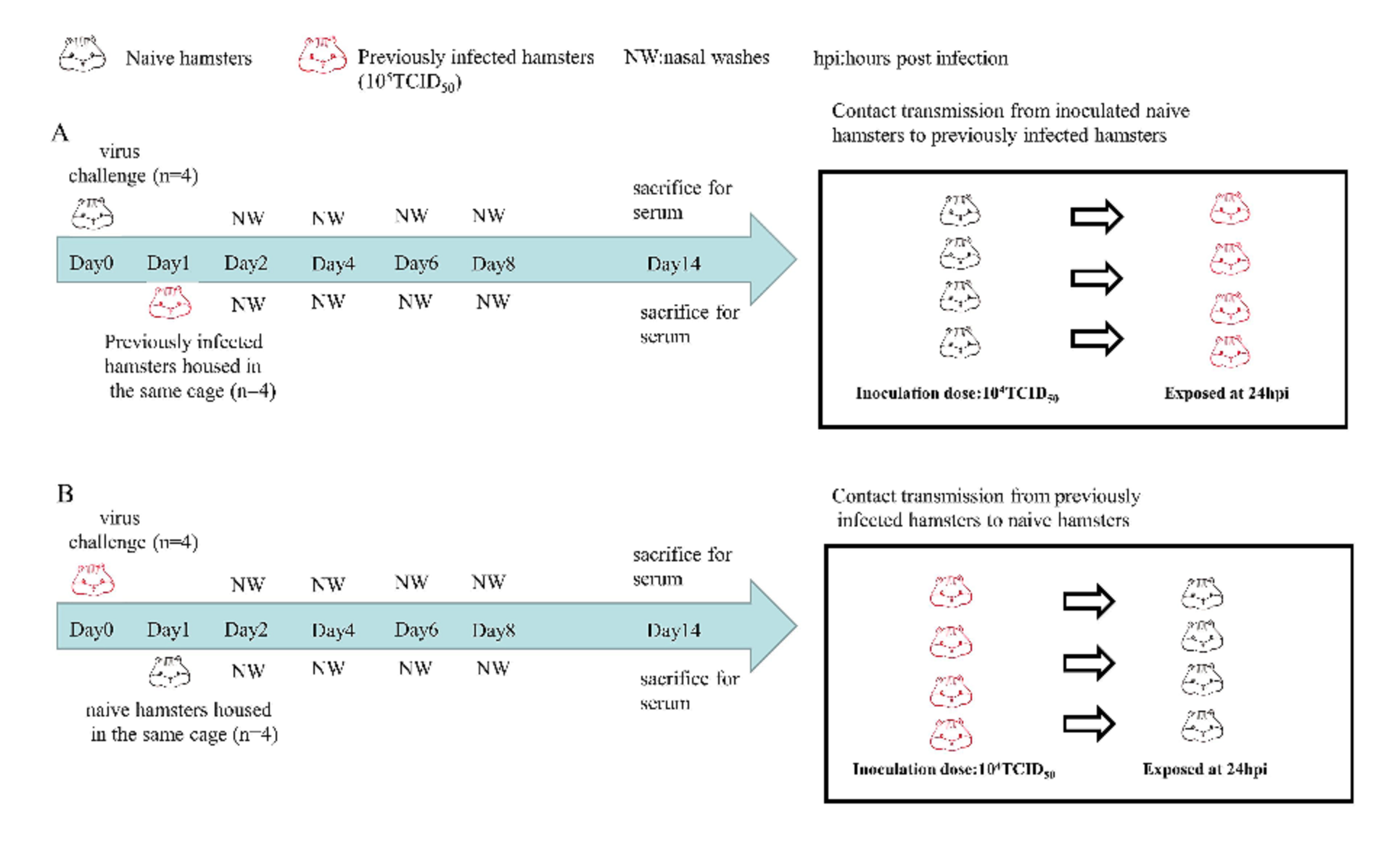


**Figure S5** The flowchart of SARS-CoV-2 transmission between naïve syrian hamsters and previously infected Syrian hamsters with a lower inoculation dose. (A) SARS-CoV-2 contact transmission from initially infected naïve Syrian hamsters (n=4) to previously infected Syrian hamsters (n=4) 24 hours after inoculation. (B) SARS-CoV-2 contact transmission from previously infected Syrian hamsters (n=4) to naïve Syrian hamsters (n=4) 24 hours after inoculation.
